# A single early-in-life antibiotic course increases susceptibility to DSS-induced colitis

**DOI:** 10.1186/s13073-020-00764-z

**Published:** 2020-07-25

**Authors:** Ceren Ozkul, Victoria E. Ruiz, Thomas Battaglia, Joseph Xu, Claire Roubaud-Baudron, Ken Cadwell, Guillermo I. Perez-Perez, Martin J. Blaser

**Affiliations:** 1 Department of Pharmaceutical Microbiology, Faculty of Pharmacy, Hacettepe University, Sihhiye, Ankara, Turkey; 2 Departments of Medicine and Microbiology, New York University School of Medicine (NYUSM), New York, NY 10016 USA; 3 Department of Biology, St. Francis College, Brooklyn, New York USA; 4 CHU Bordeaux, Pôle de Gérontologie Clinique, Bordeaux, France; 5 INSERM, UMR1053 Bordeaux Research in Translational Oncology, BaRITOn, University of Bordeaux, F-33000 Bordeaux, France; 6 Kimmel Center for Biology and Medicine at the Skirball Institute, New York University School of Medicine, New York, NY 10016 USA; 7 Department of Microbiology, New York University School of Medicine, New York, NY 10016 USA; 8 Division of Gastroenterology and Hepatology, Department of Medicine, New York University Langone Health, New York, NY 10016 USA; 9 Center for Advanced Biotechnology and Medicine, Rutgers University, New Brunswick, NJ USA

**Keywords:** DSS-induced colitis, Gastrointestinal microbiota, Pulsed antibiotic treatment, Macrolide, Childhood antibiotic use

## Abstract

**Background:**

There is increasing evidence that the intestinal microbiota plays a crucial role in the maturation of the immune system and the prevention of diseases during childhood. Early-life short-course antibiotic use may affect the progression of subsequent disease conditions by changing both host microbiota and immunologic development. Epidemiologic studies provide evidence that early-life antibiotic exposures predispose to inflammatory bowel disease (IBD).

**Methods:**

By using a murine model of dextran sodium sulfate (DSS)-induced colitis, we evaluated the effect on disease outcomes of early-life pulsed antibiotic treatment (PAT) using tylosin, a macrolide and amoxicillin, a beta-lactam. We evaluated microbiota effects at the 16S rRNA gene level, and intestinal T cells by flow cytometry. Antibiotic-perturbed or control microbiota were transferred to pups that then were challenged with DSS.

**Results:**

A single PAT course early-in-life exacerbated later DSS-induced colitis by both perturbing the microbial community and altering mucosal immune cell composition. By conventionalizing germ-free mice with either antibiotic-perturbed or control microbiota obtained 40 days after the challenge ended, we showed the transferrable and direct effect of the still-perturbed microbiota on colitis severity in the DSS model.

**Conclusions:**

The findings in this experimental model provide evidence that early-life microbiota perturbation may increase risk of colitis later in life.

## Background

Intestinal microbial colonization in early life is increasingly being connected to immune cell development [1]. The nature of the early-life maturation of the microbiome and immune system together appear to have long-lasting consequences on host physiology [2–4]. Clinical studies also have suggested the relationship of early-life gut microbiota with progression and severity of inflammatory conditions such as allergies, asthma, and inflammatory bowel disease (IBD) [5–7].

Antibiotics are the most widely prescribed therapeutic agents in children both in the USA and European countries [8–10]. Broad-spectrum beta-lactams and macrolides are the most prescribed classes in childhood [9, 11]. The majority of antibacterial drugs prescribed to children are for the treatment of common pediatric conditions such as upper respiratory tract infections, pharyngitis, and bronchitis that largely do not benefit from antibiotic therapy [12]. Studies now have shown that early-life antibiotic exposure can perturb the gut microbiota, which may further affect host physiology and health status [3, 6, 13–16].

Since microbial composition is affected by environmental factors during the critical window of early development, we asked whether early-life antibiotic exposure may have effects on the progression of subsequent disease conditions due to effects on both microbial populations and on host immunologic development. We used a murine model of the dextran sodium sulfate (DSS) challenge which experimentally induces colitis [17], to provide an indicator of the effects of tylosin, a macrolide or amoxicillin, a beta-lactam when administered early-in-life. Here we show that a single early-life antibiotic course exacerbated the colitis induced when mice were later challenged with DSS and we explored the period and mode of susceptibility.

## Methods

### Mice

Seven-week-old C57BL/6 breeding pairs were purchased from Jackson Laboratories (Bar Harbor ME) and bred in the NYUMC animal facility. Litters were randomly assigned to an experimental group and weaned at 3 weeks of age. Two to three litters were assigned to each treatment group with a target sample size of 5–11 mice per group/sex. Each treatment group included mice from separate litters to eliminate the possible differences within each litter due to different exposure to antibiotics by chance. Germ-free (GF) mice were bred in isolators at NYUMC and were conventionalized with a PAT-exposed or control inoculum of cecal contents at 6 weeks of age and followed for 14 days. Mice were maintained on a 12-h light/dark cycle and fed a standard 1% kcal fat rodent chow (PicoLab Rodent Diet 20; Brentwood, MO) and allowed ad libitum access to food and water. All mouse experiments were approved by the New York University School of Medicine Institutional Animal Care and Use Committee (IACUC protocol no. 160613) and complied with federal and institutional regulations.

### Antibiotic exposures

Treatment regimens were provided as described [18]. The antibiotic concentrations for tylosin and amoxicillin were calculated to provide 50 mg of tylosin or 25 mg of amoxicillin per kg body mass per day based on the known daily water consumption of 150 mL per kg body mass (15% of body weight/day) [18]. Briefly, tylosin tartrate or amoxicillin trihydrate (Sigma Aldrich, St. Louis, MO) were dissolved in non-acidified water at concentrations of 333 mg/L or 167 mg/L, respectively. Control mice were provided with non-acidified water. Mice were exposed at day 5 of life for 5 days, through their mothers’ milk, as described [16]. For the transfer experiment, mice were exposed to the same single tylosin course at day 5 for 5 days, and P40 cecal contents (30 days after PAT exposure ended) were collected for transfer into GF recipients.

### Induction of dextran sodium sulfate (DSS)-induced colitis and assessment of clinical disease

Since the DSS phenotype was sufficiently robust with reproducible results in each experiment [17], we used a DSS-induced colitis model in order to assess the disruptive effect of antibiotics on disease severity. DSS (molecular weight 36,000–50,000, MP Biomedicals, Solo, OH) was dissolved in water at a final concentration of 2.0% (w/v) and given ad libitum for 7 consecutive days, followed by regular drinking water for 3 to 4 days. The bottles in the cages were filled with 100 mL of water with added DSS and the water intake in each group of mice was observed daily during weight measurement experiments by measuring the remaining volume. DSS solution was administered at day 15 or 30 days after antibiotic cessation or 5 days after conventionalization with PAT-exposed or control microbiota. Animals were euthanized upon the termination of the experiment.

Mice were monitored daily during DSS challenge for weight loss, stool consistency, and stool blood using the hemoccult fecal occult blood test (Beckman Coulter, Brea, CA).

### Microbiota transfer

Transfer was performed as described [3, 16]. In brief, ceca were collected from mice that received tylosin tartrate or non-acidified water (control) between P5–10 and sacrificed at P40. The contents were divided, and 1/3 was immediately placed in pre-reduced anaerobic dental transport media (Anaerobe Systems, Morgan Hill, CA) and frozen at − 80 °C. Upon thawing under anaerobic conditions, the cecal contents were pooled and diluted in dental transport media; 100 μL of each cecal suspension was transferred to 6-week-old C57BL/6 GF mice via oral gavage. The donors were selected randomly, not from a single litter or cage, to minimize possible maternal or cage effects.

### Fecal lipocalin-2 assay

The extent of inflammation in DSS-challenged mice was assessed using the fecal lipocalin-2 (LCN-2) assay [19]. Briefly, fecal samples collected at sacrifice (P34) were reconstituted in PBS and vortex-mixed; after centrifugation, LCN-2 levels were measured in the diluted supernatants of the samples using Mouse Lipocalin-2/NGAL DuoSet ELISA kit (R&D Systems, Minneapolis, MN), according to the manufacturer’s instructions, and values determined with reference to a standard curve.

### Intestinal permeability assay

To determine intestinal permeability, mice were not fed overnight and gavaged with 4 kDa fluorescein isothiocyanate (FITC)-dextran (Sigma-Aldrich) dissolved in PBS 4 h before sacrifice, as described [17]. Blood samples were collected by cardiac puncture and immediately stored at 4 °C in the dark, serum separated, and diluted in PBS. Levels of FITC-dextran in the blood were detected by a fluorescence spectrophotometry and calculated with reference to a standard curve.

### Histopathology

Colonic tissue was collected 2 days after the end of the DSS challenge and placed into histological cassettes via the Swiss-roll technique, fixed in 10% formalin, embedded in paraffin, and processed. Hemotoxylin and eosin (H&E) staining was performed on 5-μm colon sections. Histopathological grading of inflammation and epithelial changes was performed based on the methodology by Rogers et al. [20]. Common colonic features of IBD were evaluated and scored including the degree of inflammation (1 = small multifocal lamina propria and/or transepithelial leukocyte accumulations, 2 = coalescing mucosal inflammation +/− early submucosal extension, 3 = coalescing mucosal inflammation with prominent multifocal submucosal extension +/− follicle formation, 4 = severe diffuse inflammation of mucosa, submucosa, and deeper layers), epithelium damage (1 = decreased goblet cells, occasional dilated glands, mild surface “tattering”, 2 = focally extensive surface epithelial tattering, many dilated glands with attenuated lining and luminal cell debris, 3 = erosions, 4 = ulceration), atrophy (1 = 5–25%, 2 = 25–50%, 3 = 50–75%, 4= > 75%), and dysplasia (1 = mild dysplasia, 2 = moderate dysplasia, 3 = gastrointestinal intraepithelial neoplasia, 4 = invasive carcinoma). Total histological scores and individual features were averaged per each group and statistical significance was calculated by the Mann-Whitney *U* test.

### Isolation and staining of colonic lamina propria lymphocytes

Colonic lamina propria lymphocytes were isolated using a modified method from [16]. In brief, tissues were washed in calcium/magnesium-free HBSS supplemented with 2% FCS and placed in digestion media containing 1 mM DTT and EDTA. Tissue pieces were subsequently treated with Collagenase IV/Dnase digestion mix (0.5 mg/mL of collagenase IV and 200 μg/mL Dnase). Lymphocytes were enriched using a 40%/80% discontinuous Percoll (HE Lifesciences, Pittsburgh PA) gradient. Cells were stained with LIVE/DEAD Fixable Aqua (Thermo Fisher Scientific, Waltham, MA) and the following antibody/fluorophore combination TCRb-APC, CD4-V500, (BD Bioscience, San Jose, CA) CD19-APC-Cy7, Foxp3-PECy7, Rorgt-PE (affimetrix eBioscience, San Diego, CA) and fixed with fix/perm (Affimetrix eBioscience, San Diego, CA), were used according to manufacturer’s instructions. Cells were acquired on an LSRII flow cytometer (BD Bioscience, San Jose, CA) and analyzed with FlowJo software (Tree Star, Ashland OR), with > 100,000 events collected for each sample, excluding samples with yields < 10,000 viable events.

### Gene expression in colonic tissues

RNA from harvested colonic tissues was extracted using the miRNeasy Mini Kit (QIAGEN, Hilden, Germany). After extraction, DNase digestion was done by using DNA-free DNase Treatment and Removal Reagents (Thermo Fischer Scientific, Waltham, MA). To generate the cDNA, we used the Superscript First-Strand Synthesis System for RT-PCR Kit (Thermo Fisher Scientific), with 2 μg of RNA for each sample. To detect relative expression, a parallel RT-qPCR was performed for the 18S rRNA gene [21]. Primers for TNAα [22], IL-22 [23], Muc2 [24], and Muc4 [25] were used to detect the genes of interest by RT-qPCR using in each reaction 4.0 μM of both the forward and reverse primers, in a total 20 μL reaction volume containing 1 μL of the template cDNA. The 18S, TNAα, and Muc4 cDNA samples were diluted 1:8, the IL-22 cDNA samples were undiluted, and Muc2 cDNA samples were diluted 1:2 after reverse transcription prior to qPCR. Reactions were done using the LightCycler 480 SYBR Green I Master mix (Roche) and run in a LightCycler 480 system (Roche, Indianapolis, IN). Results were analyzed using double-delta ct method comparing the relative abundance of each gene of interest to the 18S housekeeping gene [26].

### DNA extraction and library preparation

To observe changes in microbial communities, fecal samples were collected from experimental groups at specified time points. DNA was extracted from fecal or colonic samples using the Mobio 96-well extraction kit following the manufacturer’s instructions (MoBio Laboratories Inc., Carlsbad, CA). For amplicon library construction, the V4 region of the 16S rRNA gene was amplified with barcoded fusion primers [27]. Amplicons were prepared in triplicate, pooled, and quantified. The 254 bp V4 region was sequenced using the Ilumina MiSeq 2x150bp platform.

### Microbial community analysis

The Quantitative Insights Into Microbial Ecology (QIIME) program 1.90 was used to analyze data. Sequences were quality filtered and chimeras were removed. Filtered reads were clustered into 97% identity OTUs using UCLUST, followed by taxonomic assignment. Alpha diversity was calculated to determine the differences within microbial community (richness, evenness, phylogenetic diversity). The phylogenetic tree and abundance tables generated were used to calculate unweighted and weighted UniFrac β-diversity indices. Relative taxa abundances were also determined.

### Statistical methods

Significant differences in alpha diversity between experimental groups were determined using a non-parametric *t* test with 1000 permutations, while differences in β-diversity were tested by permutational MANOVA [28]. Significant differences in relative abundance were assessed using linear discriminant analysis effect size (LEfSe) [29] with *p* value < 0.05 and LDA score > 2. Two-way ANOVA and Kruskal-Wallis tests were used for multiple comparisons when appropriate. Student’s unpaired *t* test was used to compare means between groups in the germ-free mouse experiments [30].

## Results

### Effects of a single early-life antibiotic course on the severity of experimental colitis

**Fig. 1:**
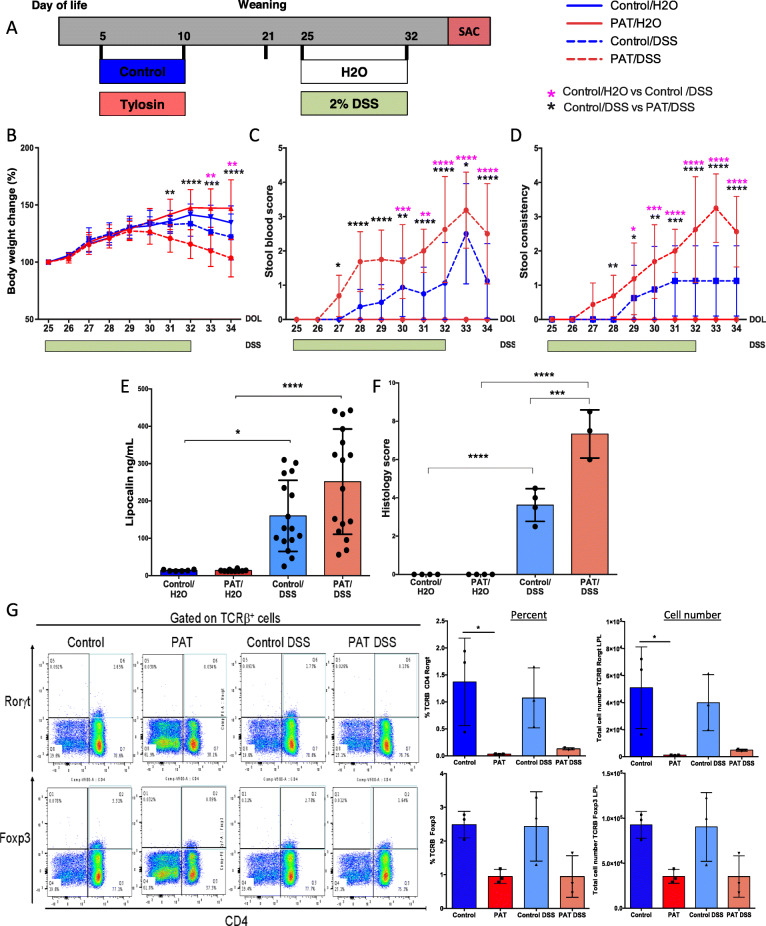
Effect of early-life antibiotic exposure on the severity of DSS-induced colitis. **a** Schematic of early DSS experiment, using a single 5-day antibiotic course (PAT). C57BL/6 mouse study groups were control/H2O (*n* = 15), PAT/H2O (*n* = 16), control/DSS (*n* = 16), and PAT/DSS (*n* = 16). Nursing dams received either tylosin or non-acidified drinking water when their pups were between 5 and 10 days old (P5-P10), and pups were exposed to tylosin or not through their mother’s milk. Experimental colitis was induced at P25 by adding 2% DSS to the pup’s drinking water or not for 7 days, and mice were sacrificed at P34. **b** Normalized percent weight decrease between the groups, measured from P25, the first day of the DSS challenge. **c** Presence of blood during and after the DSS challenge was scored as 0 (no blood), 1 (hemoccult positive), 2 (hemoccult positive and visual pellet bleeding), or 4 (gross bleeding, blood around the anus). **d** Stool consistency during and after the DSS challenge was scored as [0 (normal), 2 (loose stool), or 4 (diarrhea)] **e** Fecal lipocalin-2 levels (ng/mL) at P34; control/H2O (*n* = 6), PAT/H2O (*n* = 12), control/DSS (n = 16), and PAT/DSS (n = 16). **f** Histology scores (total of inflammation, epithelium damage, atrophy, and dysplasia scores); control/H2O (*n* = 4), PAT/H2O (*n* = 4), control/DSS (n = 4), and PAT/DSS (*n* = 3) **g** Colonic lamina propria Th17 and Treg cells shown as absolute cell numbers and as percent of total CD4 cells; control/H2O (*n* = 3), PAT/H2O (*n* = 3), control/DSS (*n* = 3), and PAT/DSS (*n* = 3). Two-way ANOVA, Kruskal-Wallis non-parametric test and Dunn’s multiple comparison testing were used for multiple comparisons. *p < 0.05; **p < 0.01; ***p < 0.001; ****p < 0.0001

Epidemiologic studies have shown strong associations with early-life antibiotic use in children and the development of IBD [6, 31]. We aimed to determine whether exposure to a single antibiotic course early-in-life would increase the severity of the experimental colitis induced in mice by DSS challenge. Nursing dams were given a pulsed antibiotic treatment (PAT) using therapeutic doses of the macrolide antibiotic, tylosin, in their drinking water from day 5 to 10 of life of their pups, as we have described [16, 32, 33]. The pups were exposed to the antibiotic in the milk ingested from their mother. At P25, after the pups had been weaned, they were given DSS in their drinking water for 7 days; clinical consequences were assessed by evaluating weight change, fecal blood, and stool consistency scores and summarized by the disease activity index (DAI) (Fig. 1a–d; Additional file 1: Fig. S1A-G). Without the DSS challenge, mice remained clinically well, whether or not they received PAT. However, in the mice receiving DSS, the PAT-exposed group had significantly more weight loss than the control group beginning 1 day before the end of DSS (P31) continuing until the end of the experiment (P34). An effect of DSS alone on body weight was observed beginning at P33, the day before the end of the experiment (Fig. 1b). Male mice were more susceptible to DSS challenge, as previously reported [34]. However, with PAT exposure, the sex effects converged (Additional file 1: Fig. S1G). Levels of fecal blood also were significantly higher in the PAT/DSS group, beginning on day 2 of the DSS challenge and continuing until their planned sacrifice on P34 (Fig. 1c). The PAT/DSS group also had significantly less stool consistency than the control/DSS group (Fig. 1d). Overall, DAI scores were significantly higher in the PAT/DSS group. All disease parameters revealed that signs for DSS-colitis severity were observed earlier in PAT-exposed mice compared to non-exposed DSS-challenged mice. As expected [17], colon length was significantly decreased in the DSS-challenged mice compared to unchallenged animals (Additional file 1: Fig. S1B); however, this was independent of PAT.

Next, we assessed the extent of intestinal inflammation, by quantitating the innate immune protein, lipocalin-2 (LCN-2), in fecal samples. The DSS-challenged groups had significantly higher LCN-2 levels than non-challenged groups; however, PAT had no significant added effect (Fig. 1e). The severity of colitis also was assessed through histopathological analysis. Among the DSS-challenged mice, those exposed to PAT (PAT/DSS) had significantly greater scores for colonic inflammation, epithelial defects, atrophy, and dysplasia than those unexposed (control/DSS) (Fig. 1f, Additional file 1: Fig. S1A). With blinded evaluation of the colon for apoptotic cells, PAT/DSS mice had significantly higher TUNEL scores compared to other groups (see Additional file 1: Fig. S1C and S1E, Additional file 2). Overall, these findings indicate that early-life exposure to tylosin exacerbated colitis induced by DSS challenge beginning 15 days after the antibiotic exposure was completed.

### Effect of early-life PAT and the DSS challenge on colonic T helper cells and mucosal gene expression

Since antibiotic-induced microbial alterations are known to alter intestinal immune populations [3, 16, 18, 35, 36], we sought to evaluate the role of the PAT exposure and the DSS challenge on colonic lamina propria lymphocytes. As observed previously [16], lamina propria Th17 cells were significantly lower in the PAT-exposed mice, and Treg cells trended lower; we now show that the DSS challenge has no added effects (Fig. 1g). These findings raise the hypothesis that the exacerbated effects of PAT on DSS-induced colitis may be related to a decrease in these TCRβ^+^ CD4^+^ Rorγt^+^ cells, but that these are not sufficient for the effect, since there was no colitis in the absence of DSS.

**Fig. 2:**
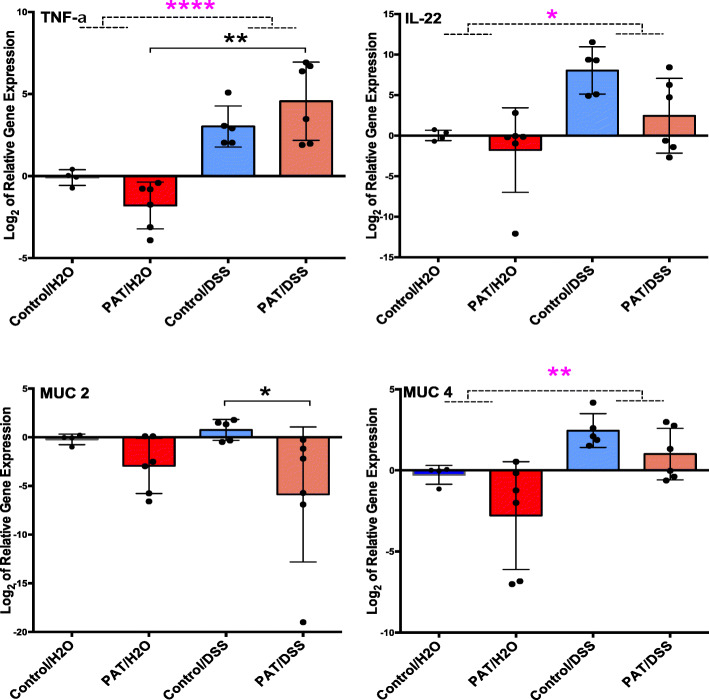
Effect of PAT and DSS-challenge on colonic gene expression. Colonic samples were obtained at sacrifice from the mice in Fig. 1, and RNA extracted. RT-qPCR was performed using primers for four genes affected by the inflammatory process (TNFα; IL-22; Muc2; Muc4). Group sizes were control/H2O (*n* = 4), PAT/H2O (*n* = 6), control/DSS (*n* = 5), and PAT/DSS (*n* = 6). Groups were also collapsed into DSS− (*n* = 10) and DSS+ (*n* = 11) and comparisons shown by dashed lines; Mann-Whitney test for the collapsed analysis. Kruskal-Wallis non-parametric test and Dunn’s multiple comparison testing were used for multiple comparisons. *p < 0.05, **p < 0.01, ***p < 0.001, ****p < 0.0001

To assess whether the exposures had a differential effect on gene expression associated with inflammatory responses and mucin production, we examined relative RNA abundances of four genes reflecting inflammatory responses and mucin production in colonic tissues at sacrifice (P34) (Fig. 2). To assess the inflammatory responses, our primary focus was on pro-inflammatory cytokines TNF-alpha and IL-22 due to their high impact on disease onset and progression in experimental colitis [37, 38], role in exacerbating inflammation in IBD (TNF-alpha) [39], and importance in intestinal wound healing and prevention of tissue damage in IBD (Il-22) [40]. We also assessed the main secretory mucin in the gut (Muc2), and a transmembrane mucin (Muc4) on the surface of intestinal epithelial cells, considering their importance both in experimental colitis and roles in postnatal intestinal mucus layer regulation related to microbial colonization [41]. The DSS challenge significantly increased expression of all genes, except Muc2, confirming the expected DSS effects on colonic inflammatory gene expression. For the mice that also were exposed to PAT, expression of TNF-α trended higher while IL-22 trended lower. In contrast, exposure to PAT alone significantly decreased Muc2 expression, and more so in conjunction with DSS. Overall, the data indicate that, as expected, the DSS challenge led to increased expression of markers of inflammation, but the prior antibiotic exposure mainly affected the expression of Muc2, involved in mucosal protection from tissue injury.

### Effect of PAT exposure and the DSS challenge on the intestinal microbiota

**Fig. 3:**
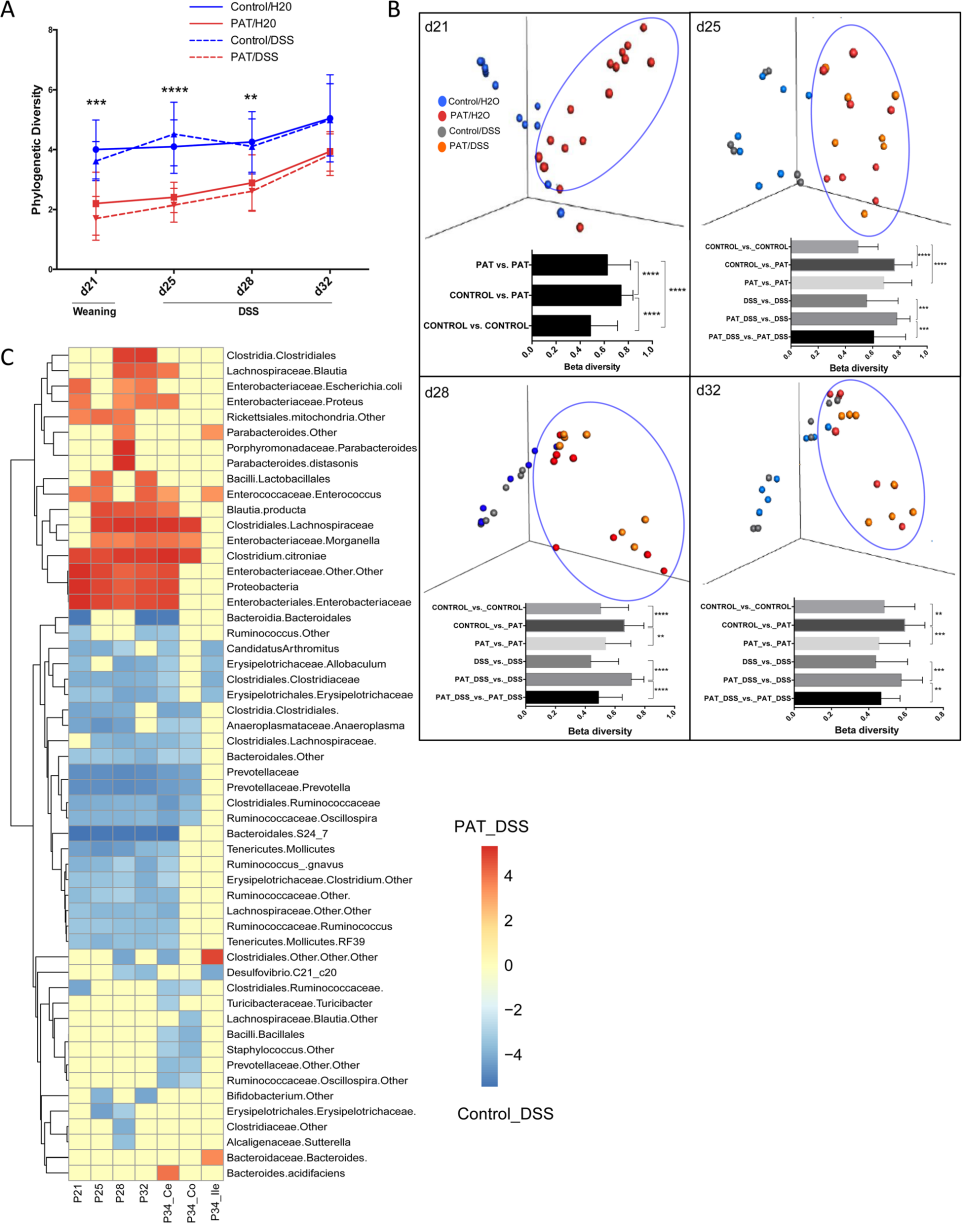
Effect of PAT and DSS-challenge on the intestinal microbial community. **a** Phylogenetic diversity (PD) scores over time. Group sizes were control/H2O (*n* = 7), PAT/H2O (*n* = 8), control/DSS (*n* = 7), and PAT/DSS (*n* = 8). **b** Comparison of microbial community structure between PAT and control groups at P21 (weaning), and between all groups at P25 (before start of DSS), P28, and P32 (end of the DSS challenge) based on unweighted UnifFrac distances as visualized by PCoA. Intra- and inter-group mean pairwise UniFrac distances are shown as bars. Intergroup community distances remain significantly greater than from P21 until day P32 for both the control and PAT-exposed groups. **c** Heat map showing significantly different taxa between the control/DSS (blue) and PAT/DSS (red) groups, using the linear discriminant analysis Effect Size (LEfSe) tool. Samples were from feces (P21, P25, P28, P31), or from the cecum (ce), colon (co), or ileum (il) at P34 sacrifice. Two-way ANOVA and Kruskal-Wallis tests were used for multiple comparisons. *p < 0.05, **p < 0.01, ***p < 0.001, ****p < 0.0001

Next, we examined the effects of the PAT (tylosin) exposure and the DSS challenge on intestinal microbial communities. We collected fecal pellets at weaning and 1, 3, and 7 days after the DSS challenge was begun, as well as ileal, colonic, and cecal samples at sacrifice. Intestinal microbial community diversity was significantly decreased in the PAT-exposed mice (Fig. 3a). Microbial richness and evenness was significantly decreased in PAT-exposed mice compared to the controls at weaning (P21, 11 days after PAT), persisting until P32 (3 weeks after cessation of the exposure) (Additional file 1: Fig. S1D). While PAT effects on colonic and cecal populations were comparable to those in the fecal samples, ileal community differences were less (data not shown), in accordance with prior literature indicating the highly dynamic [42] and less diverse [43] nature of the small intestinal microbiota. The DSS challenge had no significant effect on the microbial richness.

Microbial community structure (β-diversity) was significantly distinct between groups starting from P21, continuing until P32 (final day of DSS), according to UniFrac analysis. At baseline (P21 (pre-DSS)), the PAT and control groups were significantly different from each other. After the DSS challenge was begun, the significant differences between the PAT and control groups remained, while DSS had no significant effect (Fig. 3b). These results confirmed the distinct continuing effects of PAT on the intestinal microbial communities. There were no gender-specific differences in community richness, evenness, and structure. Independent from any DSS effect, relative taxa abundances also were distinct between PAT and control mice (Additional file 1: Fig. S1E). Over the course of the experiment, fecal microbial communities between PAT and control groups remained distinct.

Linear discriminant analysis effect size (LEfSe) analysis revealed that the S24-7 family, Prevotellaceae, and multiple other taxa were significantly more abundant in control microbiota compared to PAT at baseline, continuing after the DSS challenge. In contrast, significantly increased and continuing abundances of *Clostridium citroniae* and Enterobacteriaceae were observed in PAT (Fig. 3c). The DSS challenge had few additional effects.

### Effects of early-life amoxicillin exposure

We next directly compared the effects of the amoxicillin, tylosin, and control exposures. In the amoxicillin-exposed mice, the DSS-induced colitis was less severe than in mice exposed to tylosin. There were no significant differences in weight change, fecal blood excretion, and histologic scores between the amoxicillin-exposed and the unexposed mice (Additional file 1: Fig. S2A-F). Both alpha diversity and microbial community structure were not significantly different from control unlike the distinct tylosin effects (Additional file 1: Fig. S2G-H). Taxa abundances and LEfSe analysis revealed minor taxonomic differences between the amoxicillin and control groups (Additional file 1: Fig. S2I, and not shown). This, as in prior studies comparing the effects of amoxicillin and tylosin PAT [18, 33], the amoxicillin-induced disturbance was less, paralleling findings in antibiotic-exposed school children [44].

### Persistence of effects from early-life tylosin exposure on colitis severity

**Fig. 4:**
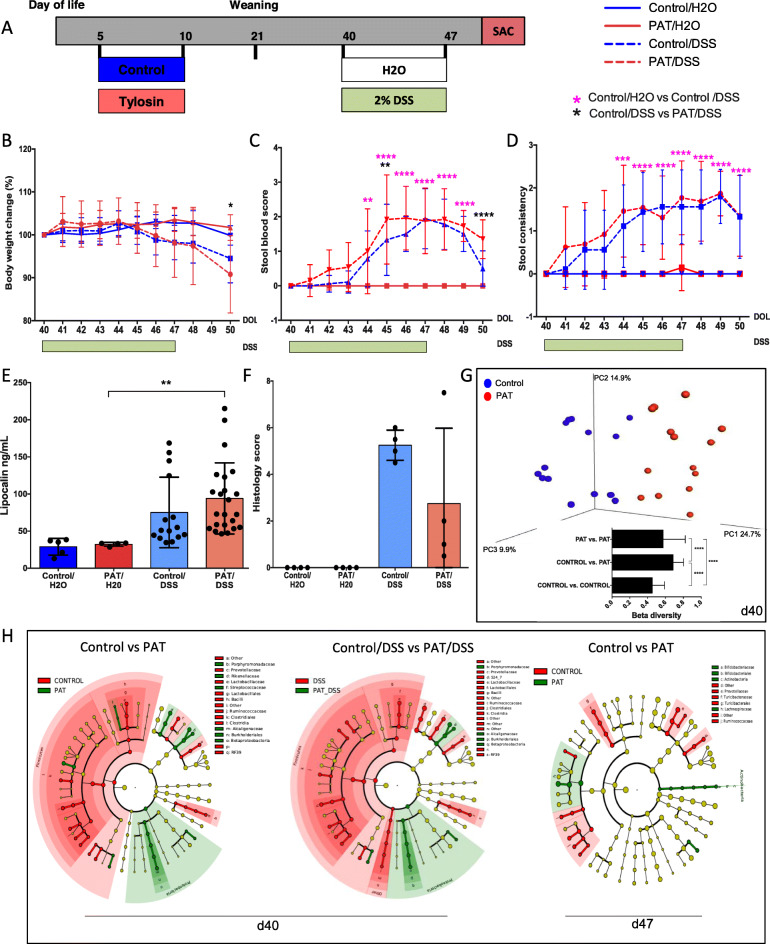
Lasting effects of early-life antibiotic exposure on DSS-induced colitis severity and on intestinal microbial communities. **a** Schematic of the late time point DSS experiment. Sample sizes were the control/H2O (*n* = 9), PAT/H2O (*n* = 14), control/DSS (*n* = 18), and PAT/DSS (*n* = 26) mice for **a**–**d**. For lipocalin analyses (**e**) sample sizes were the control/H2O (*n* = 5), PAT/H2O (*n* = 4), control/DSS (*n* = 15), and PAT/DSS (*n* = 23). For histology scoring (**f**) sample sizes were the control/H2O (*n* = 4), PAT/H2O (*n* = 4), control/DSS (*n* = 4), and PAT/DSS (*n* = 4). For microbiome analyses (**g**, **h**) sample sizes were the control/H2O (*n* = 6), PAT/H2O (*n* = 8), control/DSS (*n* = 8), and PAT/DSS (*n* = 8). Tylosin exposure or not and study design was exactly as in Fig. 1, except experimental colitis by the DSS challenge was induced at P40, 30 days after PAT ended instead of P25. **b**–**f** used the same measurements and criteria as in Fig. 1, except at the time points reflecting the different study design. Fecal lipocalin-2 levels were measured at P50. **g** Unweighted UniFrac distances between the PAT and control groups at P40 (before start of DSS), and mean pairwise UniFrac distances within and between groups. **h** LEfSe cladograms indicating significantly differential taxa in control and PAT mice at P40 (30 days after PAT and immediately before the DSS challenge) and P47 (following the DSS challenge). Two-way ANOVA and Kruskal-Wallis tests were used for multiple comparisons. *p < 0.05, **p < 0.01, ****p < 0.0001

To determine whether the effects of the early-life (P5–10) tylosin-PAT exposure in relation to colitis were persistent, we next challenged mice with DSS 30 days (P40) after the exposure period had ended (Fig. 4a). There were no significant differences between the groups at P40, prior to the challenge. Both body weight effects and stool blood scores became more severe in the PAT-exposed group, compared to the unexposed DSS group at the time of sacrifice (P50) (Fig. 4b, c). No significant difference was observed in stool consistency scores between the PAT-exposed and unexposed DSS groups (Fig. 4d). There were no significant differences in histologic scores or in fecal LCN-2 levels in the DSS-challenged mice, whether or not they received the PAT exposure 30 days earlier. Thus, the clinical effect of the prior antibiotic exposure attenuated over time (Fig. 4a–f).

### Effect of tylosin treatment on the microbial community in the later time period

Next, we examined the duration of the effects of the early-life tylosin exposure on the microbiota. By P40, microbial richness and evenness (alpha-diversity) began increasing, becoming similar to the controls (data not shown). However, the microbial community structure of the PAT and control mice remained distinct at P40 (Fig. 4g). As described above, the short-term DSS challenge had no substantial effect on intestinal microbiota composition (data not shown). As before, Proteobacteria and Rikennellaceae were abundant, and Prevotellaceae, S24–7, and Ruminococcaceae were significantly decreased in the PAT mice at weaning. By P40, Proteobacteria and Rikennellaceae remained significantly more abundant in PAT mice. By P47, PAT and control compositions largely converged; however, Ruminococcaceae remained significantly decreased in PAT mice (Fig. 4h). In total, although diminishing over time, the PAT effects on the microbiota persisted for weeks.

### The PAT-altered microbiota is sufficient to enhance DSS-induced colitis

**Fig. 5:**
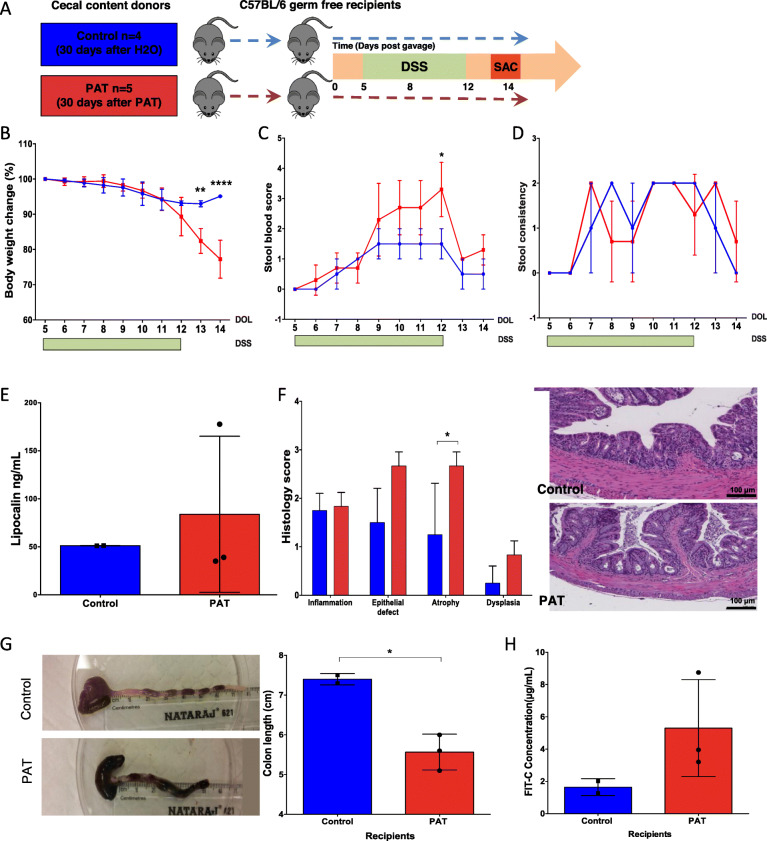
Effect of transferring PAT-altered microbiota to germ-free mice on DSS-induced colitis and intestinal microbial community. **a** Schematic of the transfer experiment. Six-week old germ-free (GF) mice were gavaged with either PAT-perturbed (*n* = 5) or control (*n* = 4) cecal contents from P40 donor mice (30 days after the end of the PAT or control exposure). The now-conventionalized recipient mice (3 PAT and 2 control) were challenged with DSS 5 days after the cecal transfer. **b**–**e** Used the same measurements and criteria as in Fig. 1, except at the time points reflecting the different study designs. **f** Histology of the colon in control and PAT recipients. Magnification × 10, H&E staining. Individual scores for inflammation, epithelial injury, atrophy, and dysplasia. **g** Representatives of differences in mean colon length between control and PAT recipients. **h** Intestinal permeability measured by fluorescein isothiocyanate (FITC)-dextran in blood. Two-way ANOVA and unpaired *t* test were used for comparisons. *p < 0.05, **p < 0.01, ****p < 0.0001

We next sought to determine whether the persistently altered microbiota played a direct role in the heightened susceptibility to DSS colitis. To that end, from P40 donors who were either exposed to PAT (30 days after exposure ended) or were control (not exposed), we transferred their cecal contents to 6-week-old C57BL/6 GF mice. The recipient mice then were challenged with DSS 5 days after the cecal transfer (Fig. 5a). Body weight decreases became significantly greater in the recipients of the PAT-altered microbiota by day 13 post-gavage (1 day after the DSS challenge ended) (Fig. 5b). Fecal blood scores tended to be higher in the recipients of the PAT-altered microbiota and significantly higher at the end of the DSS challenge in the mice exposed to the PAT-perturbed microbiota (Fig. 5c). No significant difference was observed in stool consistency and lipocalin levels (Fig. 5d, e). Recipients of the perturbed microbiota had significantly more atrophy (*p* = 0.02), trended toward higher overall histology scores (4.5 ± 1.5 vs. 8.0 ± 0.5; *p* = 0.07) (Fig. 5f), had significantly shorter colon length (Fig. 5g), and trended toward higher intestinal permeability than control recipients (Fig. 5h), These findings provide evidence that even 30 days after the antibiotic exposure ended, the perturbed microbiota per se is sufficient to worsen the DSS-induced colitis.

### Alteration in microbial community diversity and structure after conventionalization of germ-free mice with PAT or control microbiota

The pools used to conventionalize mice with either the control or PAT-perturbed microbiota were similar to the individual constituent mice in each group (Additional file 1: Fig. S3A). Compositions in the PAT and control recipient mice remained distinct over the study period, reflecting the distinct compositions of the donors, with decreased abundance of Prevotellaceae, S24–7, Bacteroidia, and Ruminococcaceae in the PAT group (Additional file 1: Fig. S3B). Microbial community structure was significantly different between P40 control and PAT donors (Additional file 1: Fig. S3C), and the inocula prepared by pooling the donor cecal contents were also similar to the respective recipient fecal samples at each time point as well as the ileal, cecal, and colonic samples (Additional file 1: Fig. S3D) The recipients reflected the distinct community structures of the donor pools at days 5 and 14 post-gavage (Additional file 1: Fig. S3E), confirming the successful transfer of the microbiota. Phylogenetic (α-) diversity rose in the control microbiota recipients up to day 14 post gavage, but not in the PAT-recipient mice (Additional file 1: Fig. S3F). Compared with control, the PAT donor cecal pool had decreased S24–7 and increased *Akkermansia muciniphila*, *Bacteroides acidifaciens*, and *Clostridium citroniae* (Additional file 1: Fig. S3G), similar to the prior experiments, with specific taxonomic differences persisting over the course of the experiment. This work demonstrates that the antibiotic-perturbed microbiota was transferable and that the perturbed transferred microbiota persisted, consistent with the worsened colitis observed.

## Discussion

Our present study, designed to model a short antibiotic course in a young child, provides evidence that even a single early-life antibiotic course affects colitis severity in a model of IBD [17, 45]. Studies of human children with IBD evaluated past antibiotic exposure [15, 31, 46, 47], and their association with the development of childhood IBD [31], whereas animal studies enable prospective evaluations of causal relationships.

A beta-lactam antibiotic (amoxicillin) and a macrolide (tylosin) were used, as these classes of antibiotics are the most frequently prescribed to children worldwide [9, 12]. The dosing and the course of our exposure model in mice mimic antibiotic perturbations in children as shown in prior studies [3, 13, 16, 18]. By using this model, we found that early-life antibiotic treatment altered microbial community structure and diversity as well as Th17 cell representation, leading to enhanced colitis when animals were challenged with DSS 15 days later. Also consistent with our prior studies [13, 16, 18] and the literature in children [44], the macrolide had stronger effects than the beta-lactam. One explanation for the differential effects between antibiotics may reflect their relative spectrum of activity. The macrolide, tylosin, has the same mechanism of action on protein synthesis by binding to the 50S rRNA subunit as the macrolides widely used in human children (erythromycin, clarithromycin, azithromycin). Amoxicillin inhibits bacterial cell wall synthesis and has limited effects on anaerobic bacteria [48]. Macrolides are active against Gram-positive bacteria including non-spore-forming anaerobic bacilli, with limited direct effects on Gram-negative bacteria, including Enterobacteriaceae [49]. Accordingly, we observed continuing abundance of Enterobacteriaceae in tylosin-exposed mice, similar to previous observations [16]. Our findings also are consistent with broad-spectrum antibiotic exposure effects in human infants in which Enterobacteriaceae were increased for two months [50].

After the DSS challenge, intestinal cell death as determined by TUNEL-positive cells was significantly higher in the antibiotic-exposed mice than in the control-exposed mice, suggesting effects on the induction of apoptosis, although our group sizes were small (Additional file 2). Clinical studies have shown increased epithelial apoptosis in ulcerative colitis patients [51, 52]. One hypothesis is that the antibiotic perturbed microbiota may decrease the colonic epithelial cell cytoprotective properties of specific bacterial taxa [53].

Fecal lipocalin is considered a sensitive marker for intestinal inflammation [19]. Lipocalin, expressed in several cell types, is released mainly from neutrophils, and its presence is related with both epithelial damage and neutrophil presence [54]. That PAT exposure had no significant effect on lipocalin levels after the DSS challenge may reflect the scale of the DSS-induced epithelial cell injury.

The composition of the gut microbiota is known to affect intestinal immune cell populations and inflammatory disease risk [2, 7, 16, 55–57]. In IBD [58], gut microbiota diversity and richness are substantially decreased [59], but in observational studies of humans, the causal direction is unknown [60]. In IL-10 or IL-2 deficient mice, GF animals develop milder IBD suggesting microbiota roles in the inflammatory process [61, 62].

In our study, mice with antibiotic-perturbed microbiota had significantly fewer colonic lamina propria Th17 cells and a trend to fewer Treg cells. The effect on immune cells was independent of the DSS challenge, as there was no difference between control and the DSS-challenged mice. Although our observations on T cell alterations included the post-DSS period, our previous results showed that PAT led to a decrease in Th17 cells as early as at day 27 (17 days after the antibiotic exposure ended) [16]. Together, these data suggest that the main effects seen are due to PAT exposure regardless of the DSS challenge or not. Although both T helper populations play important roles in IBD pathogenesis [59, 63, 64], our GF transfer study showed that a PAT-perturbed microbiota is sufficient for increased colitis severity. Although we did not examine the T cell populations after the microbiota transfer to GF mice, our prior study showed that a PAT-perturbed microbiota was sufficient to impair host immunological development in recipient mice for at least 77 days post-transfer [16]. Although the timing and design of the two studies differ, impaired immunological functions in conventionalized GF mice also likely are shaped by the colonizing populations, indicating a direct or indirect causal role of the perturbed microbiota.

Our late DSS-challenge experiments (at P40) revealed that the clinical effect of prior antibiotic exposure attenuated over time, despite persistence of PAT-altered microbiota. Nevertheless, the PAT-altered microbiota from P40 donors was sufficient to worsen colitis severity. Since conventionalized GF mice have increased susceptibility to intestinal inflammation [1], we hypothesize that the GF setting adds a further immunological insult to that induced by DSS, potentiating the effects of the transferred perturbed microbiota, facilitating detection.

Jin et al. also examined the relation between antibiotic exposure and subsequent DSS challenge [65]. However, their methods differed from ours in the exposure timing (day 35 vs. day 5), duration (14 vs. 5 days), antibiotics used (penicillin, metronidazole, or enrofloxacin vs. tylosin), and level of antibiotic exposure (sub-therapeutic vs. therapeutic). Although metronidazole and enrofloxacin had no significant effects, penicillin was protective, in association with diminished small intestinal Th17 populations. We also found Th17 suppression in the PAT-exposed mice, but the divergent disease phenotypes in the two studies suggest the importance of the particular antibiotics used and the timing of the exposures, as not all microbial members may have contributed equally to pathological imprinting later in adulthood [66]. Our results are consistent with those of Al Nabhani et al. [66], as both studies underscore the increased disease susceptibility due to microbiota perturbations during a critical window, and show the important Treg role in susceptibility to later-in-life inflammatory pathologies. Treg cells are critical for immune tolerance in the gut [59], and the lower levels we observed are consistent with enhanced disease [66], but that the perturbed microbiota transfer the phenotype is against a necessary role. Colonic lamina propria Treg cell numbers are diminished in GF mice [67, 68] and are lower in our study, despite opposing disease phenotypes.

The antibiotic exposure reduced abundance of the immunomodulatory segmented filamentous bacteria (Candidatus Savagella) [57] and S24-7 (Candidatus Homeothermaceae), consistent with prior findings [16, 18].

As expected, the DSS challenge led to increased expression of inflammatory genes, but the antibiotic exposure led to decreased expression of genes involved in colonocyte mucus secretion [41, 69], barrier integrity, and modulating inflammatory responses [41, 70]. However, the diminished mucus production following antibiotic exposure provides a mechanism [71] for enhanced tissue damage. Since the antibiotic exposure ended 15 days prior to the DSS challenge, the effects on gene expression most likely were not direct, but indirect, via an antibiotic-altered microbiota, These results are consistent with our prior finding that ileal gene expression profiles did not differ between PAT-exposed and unexposed GF mice, indicating lack of direct antibiotic effects [16]. Moreover, with *Citrobacter rodentium*-induced colitis, metronidazole treatment, leading to enhanced colonic inflammation and altered goblet cell function, provides another example of microbiota alteration affecting the protective mucin role [72]. Our results are consistent with the hypothesis that early-life antibiotic exposure, by perturbing the microbiota and affecting short-chain fatty acid synthesis [69, 73], decreases protective epithelial cell mucin production, leading to enhanced inflammation when challenged by a stimulus like DSS.

Studies that have shown protective effects of antibiotics have largely focused on adult mice [65, 74]. Munyaka et al. [75] studying the role of antepartum antibiotics showed enhanced DSS-colitis severity and perturbed offspring microbiota, similar to our findings, as did a study involving gavaging pregnant IL-1-deficient mice with perturbed microbiota [13].

Although delaying the DSS challenge to 30 days post-antibiotic exposure rather than 15 days led to a lessened effect, the ability of P40 cecal content to worsen DSS colitis following transfer suggests that there remain colitogenic effects of the PAT-perturbed microbiota. In the *C. rodentium*-induced colitis model, prior studies’ disease enhancement persisted 80 days after the antibiotic exposure ceased [33]. Thus, even with apparent normalization of the major microbial composition changes, differences with functional significance may remain.

We recognize the following limitations of our study. Although our study showed that early-life antibiotic-perturbed microbiota lead to increased colitis severity and altered immune cell populations, we did not test the persistence of the immune cell alterations. The lessened effect of PAT on disease severity in the late DSS-challenge experiments could be explained by the restoration of normal immune cell populations. While our prior studies showed long-lasting perturbations in T cell populations up to 42 days after PAT exposure [16], whether the exposure irrevocably altered immunological development has not yet been defined but needs to be tested in future studies. The further consequences of both immune and microbial alterations on colitis severity with more remote DSS-exposure might clarify this issue. Conventionalizing GF mice with selective enrichment of altered keystone taxa might be another approach to confirm their contributions to disease phenotypes.

## Conclusions

In conclusion, early-life antibiotic exposure exacerbated murine DSS-induced colitis and the altered microbiota was sufficient to transfer the phenotype. Early-life macrolide use may have unexpected risks.

## Supplementary information

**Additional file 1.** Supplementary figures supporting the results of this article.

**Additional file 2.** Apoptosis assay. Methods and results for apoptosis assays.

## Data Availability

The 16S sequencing data are available in QIITA with the identifier 12996 (https://qiita.ucsd.edu/study/description/12996) [76].

## Abbreviations

DSS: Dextran sodium sulfate
IBD: Inflammatory bowel disease
PAT: Pulsed antibiotic treatment
DAI: Disease activity index
LCN-2: Lipocalin-2
TUNEL: Terminal deoxynucleotidyl transferase dUTP nick end labeling
TNF-α: Tumor necrosis factor alpha
IL-22: Interleukin-22
Muc: Mucin
LEfSe: Linear discriminant analysis effect size
PCoA: Principal coordinates analysis
GF: Germ-free
FITC: Fluorescein isothiocyanate
FCS: Fetal calf serum
DTT: Dithiothreitol
PBS: Phosphate buffered saline
DAPI: 4′,6-diamidino-2-phenylindole
QIIME: Quantitative Insights Into Microbial Ecology
