# Supplementary material for: A single early-in-life antibiotic course increases susceptibility to DSS-induced colitis
Source: Genome Med. 2020 Jul 25;12:65. doi: 10.1186/s13073-020-00764-z (PMC7382806; doi:10.1186/s13073-020-00764-z)
Supplement: Supplementary file 1 — Additional file 1. Supplementary figures supporting the results of this article. [file 13073_2020_764_MOESM1_ESM.pdf]

Additional file 1: Supplementary figures

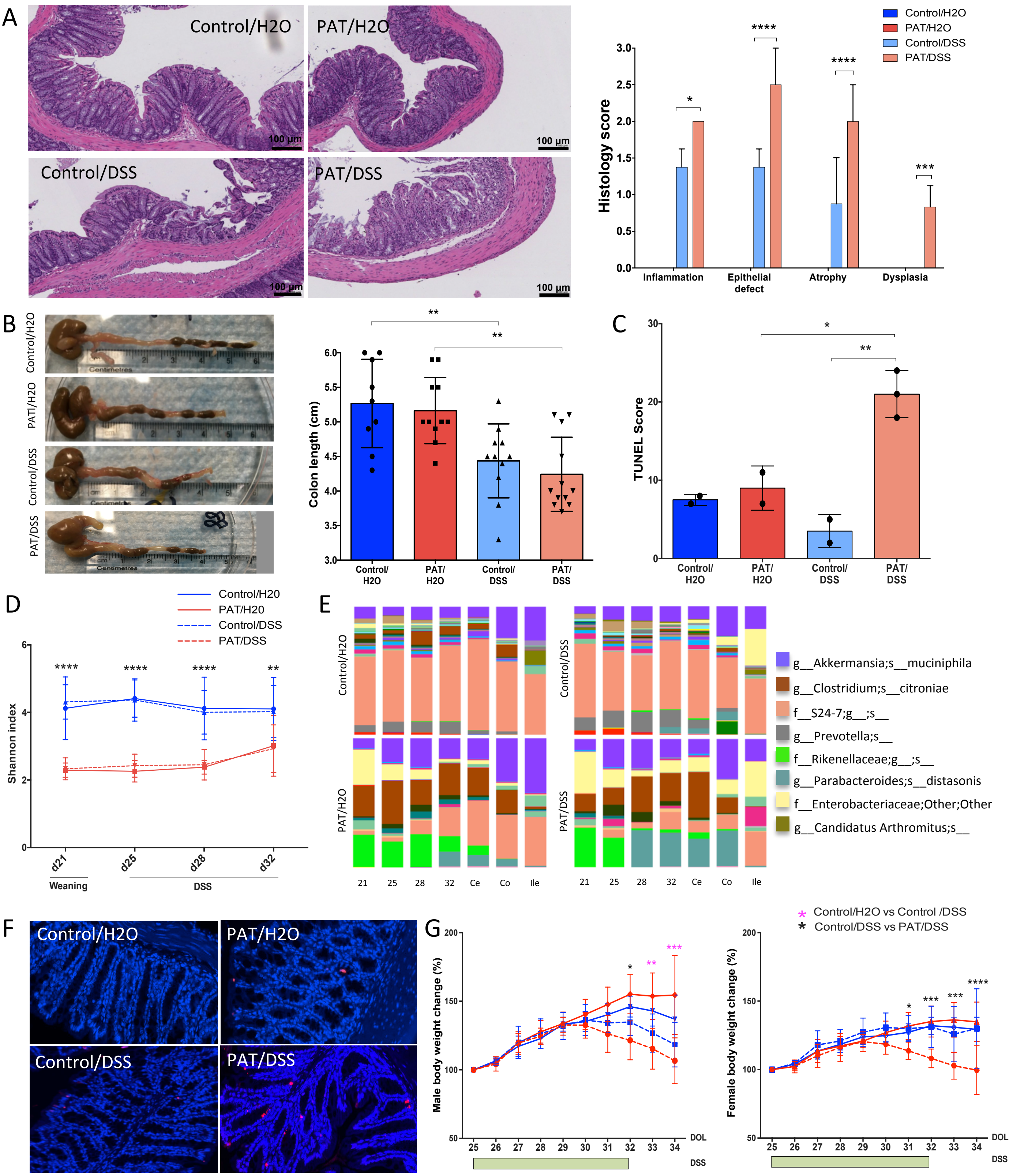

Fig. S1**:** Effect of early-life antibiotic exposure on severity of DSS-induced colitis 15 days after the course. A) Representative histology of the colon at sacrifice at P34. Magnification 10X, H&E Staining, and individual scores for inflammation, epithelial injury, atrophy and dysplasia. B) Representative differences in mean colon length between all groups. C) TUNEL assay indicating apoptotic cell numbers, blindly scored for representative mice per each group; Control/H2O (n=2), PAT/H2O (n=2), Control/DSS (n=2), and PAT/DSS (n=3). D) Shannon index between the groups over time. E) Taxa abundances over time and for each sample type. Intestinal samples are from day 34 of life (sacrifice), which is 2 days after the DSS challenge ended. F) TUNEL showing brightly-stained apoptotic nuclei in murine colon wall. G) Normalized % weight decrease between the groups by sex. Two-way ANOVA and Kruskal-Wallis tests were used for multiple comparisons. *p<0.05, **p<0.01, ***p<0.001, ****p<0.0001.

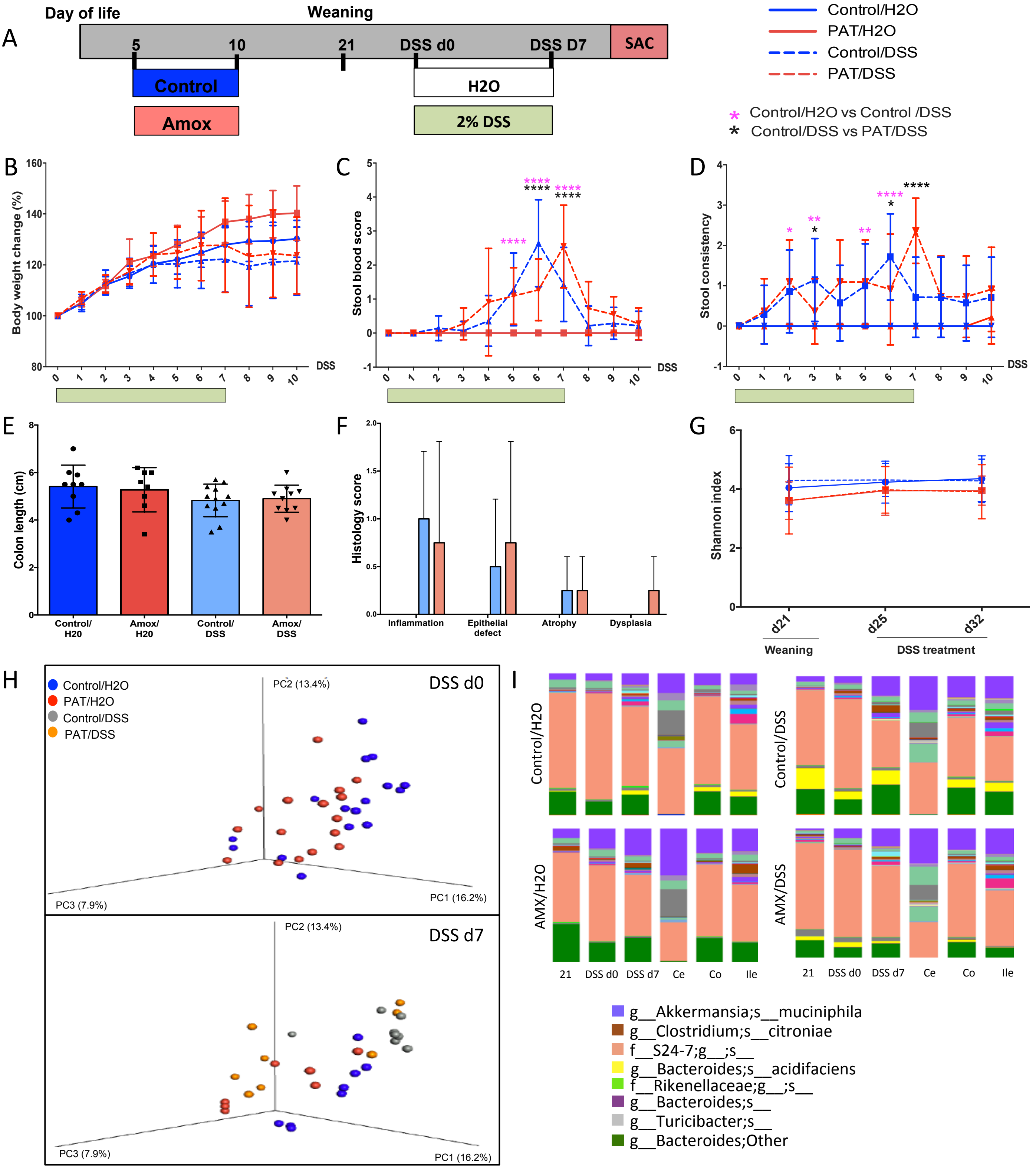

Fig. S2: Effect of single amoxicillin course on severity of DSS-induced colitis and intestinal microbiota. A) Schematic of amoxicillin experiment. Mice challenged with DSS 16-18 days after the end of amoxicillin. Start of the DSS challenge (DSS 0) at P26 and P28 in two different cohorts. Sacrifice was 3 days after the completion of DSS. Study groups consisted of Control/H2O (n=9), PAT/H2O (n=9), Control/DSS (n=14), and PAT/DSS (n=11). Panels B-D used the same measurements and criteria as in Figures 1 and 4, except at the time points reflecting the different study design. B) Normalized % weight decrease between the groups. C) Stool blood scores during and after DSS challenge between the groups. D) Stool consistency scores. E) Mean of the colon length between the groups. F) Individual histology scores for inflammation, epithelial defects, atrophy and dysplasia; Control/ H2O (n=4), PAT/H2O (n=4), Control/DSS (n=4), and PAT/DSS (n=4). For microbiome analyses (Panels G-I) sample sizes were; Control/H2O (n=8), PAT/H2O (n=8), Control/DSS (n=8), and PAT/DSS (n=8). G) Shannon index between the groups over time. H) Unweighted UniFrac distances between control and PAT recipients at start of DSS (DSS d0) and between all groups at the end of DSS (DSS d7). I) Taxa abundances over time and per sample type. Two-way ANOVA and Kruskal-Wallis tests were used for multiple comparisons. ****p<0.0001.

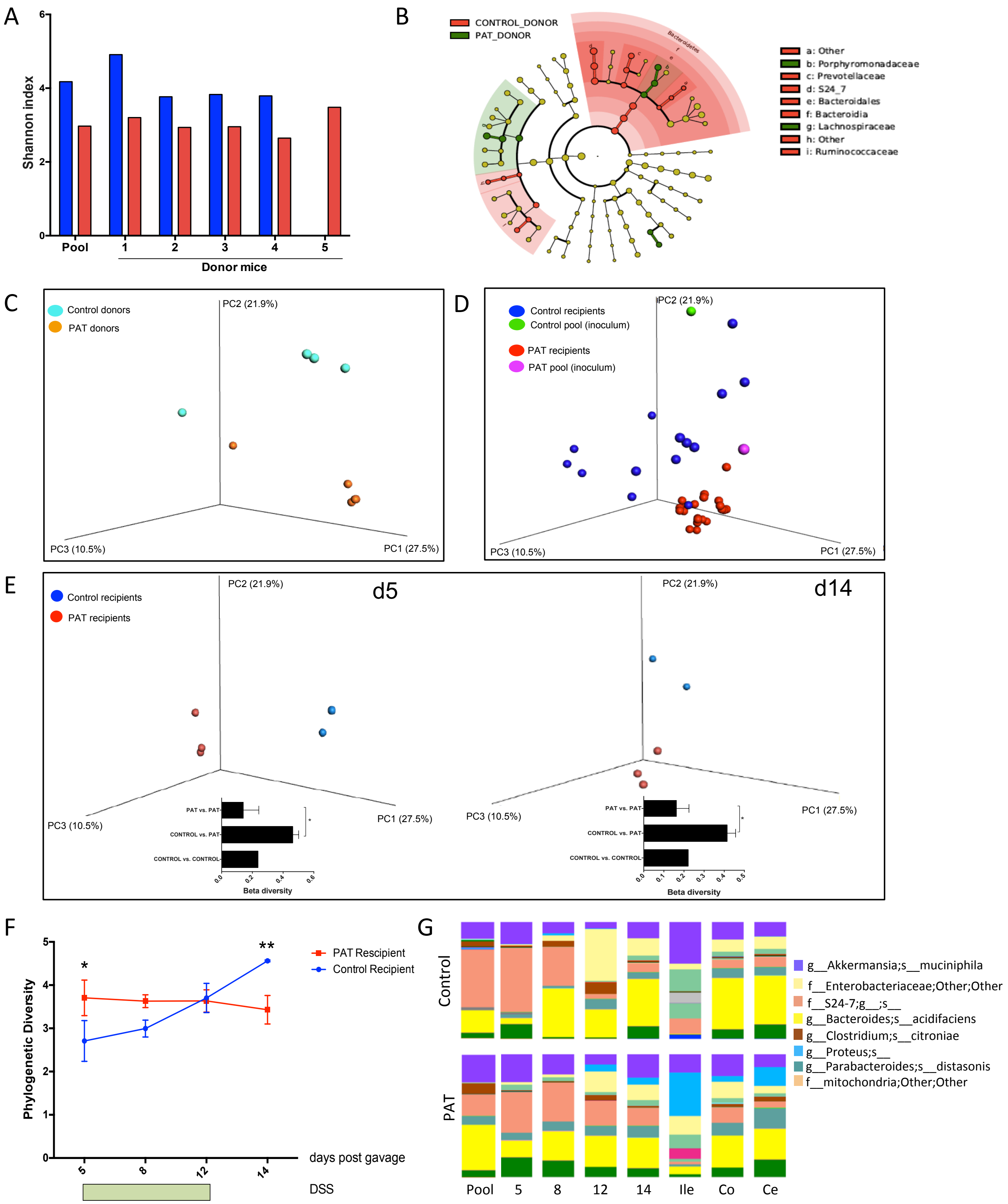

Fig. S3: Intestinal microbial community effects from transferring PAT-altered microbiota to germ-free mice. A) Shannon index of transferred cecal content pool and individual donors (4 control, 5 PAT donors). B) Significantly different taxa abundances in the Control and PAT cecal donors as determined using the Linear discriminant analysis Effect Size (LEfSe) tool. C) PCoA of unweighted UniFrac distances among individual Control and PAT donor mice at day40 (30 day after PAT exposure ended). D) PCoA of unweighted UniFrac distances for the two donor pools and among recipients (fecal samples at d5, d8, d12, d14 post-gavage and ileal, colonic and cecal contents at sacrifice). E) PCoA of unweighted UniFrac distances between control and PAT recipients at days 5 and14 days post-gavage with comparisons within and between groups F) Microbiome phylogenetic (α-) diversity (PD) over time between control and PAT recipients. G) Taxa abundances in the donor pools and in recipients day 5-14 fecal samples, and intestinal samples (ileum, cecum, colon) at sacrifice on experiment day 14. Comparisons were performed by two-way ANOVA and Kruskal-Wallis; *p<0.05, **p<0.01.
