# Supplementary material for: A single early-in-life antibiotic course increases susceptibility to DSS-induced colitis
Source: Genome Med. 2020 Jul 25;12:65. doi: 10.1186/s13073-020-00764-z (PMC7382806; doi:10.1186/s13073-020-00764-z)
Supplement: Supplementary file 2 — Additional file 2. Apoptosis assay. Methods and results for apoptosis assays. [file 13073_2020_764_MOESM2_ESM.docx]

**Additional file 2**

**Methods**

**Apoptosis Assay**

Apoptotic cells were identified by the terminal deoxynucleotidyltransferase-mediated dUTP end labeling (TUNEL) technique using *in situ* cell death detection Kit (Roche Diagnostics, TMR In Situ Cell Death Detection Kit). Briefly, formalin-fixed, paraffin-embedded murine colonic tissue sections were heated at 65°C for one hour and the TUNEL assay performed for 3 hours, according to the manufacturer’s instructions. Upon completion, slides were washed in PBS. Nuclei were counterstained in DAPI (0.5 ug/ml, 4',6-diamidino-2-phenylindole in Dulbecco’s phosphate buffered saline) for 3 minutes at room temperature, rinsed in PBS and then mounted with Prolong anti-fade (Molecular Probes, Life Technologies and Invitrogen respectively, Thermo-Fischer Scientific Waltham, MA). TUNEL-positive cells were blindly counted in five different random fields in all samples, and mean numbers for apoptotic cell counts per group were compared.

**Results**

With blinded evaluation of the colon for apoptotic cells, PAT/DSS mice had significantly higher TUNEL scores compared to other groups (vs. Control/Control, p=0.007; vs. PAT/Control, p=0.012; vs. Control/DSS, p=0.002) (Figure S1C, Figure S1F).
